# *Pancoriusguiyang* sp. nov., a new species of jumping spiders (Araneae, Salticidae) from Guizhou Province, China

**DOI:** 10.3897/BDJ.11.e108159

**Published:** 2023-08-08

**Authors:** Weicheng Yang, Yufeng Zhou, Dongxue Gu, Hao Yu

**Affiliations:** 1 The State Key Laboratory of Southwest Karst Mountain Biodiversity Conservation of Forestry Administration, School of life sciences, Guizhou Normal University, Guiyang, China The State Key Laboratory of Southwest Karst Mountain Biodiversity Conservation of Forestry Administration, School of life sciences, Guizhou Normal University Guiyang China; 2 School of Biological Sciences, Guizhou Education University, Guiyang, China School of Biological Sciences, Guizhou Education University Guiyang China

**Keywords:** new species, morphology, DNA barcoding, diagnosis, taxonomy

## Abstract

**Background:**

*Pancorius* Simon, 1902 is a relatively large genus of jumping spider family Salticidae and currently contains 42 valid species that are mainly distributed in South East Asia, 11 of which are recorded from China.

**New information:**

A new spider species of the genus *Pancorius* from Guiyang City in southwest China, is described under the name of *P.guiyang* Yang, Gu & Yu, sp. nov. Detailed descriptions and photographs are provided. DNA barcodes (a partial fragment of the mitochondrial cytochrome oxidase subunit I gene, COI) of the species were obtained to confirm matching of the sexes and for future use in molecular studies.

## Introduction

*Pancorius* Simon, 1902 is a relatively large spider genus of family Salticidae Blackwall, 1841, with a restricted distribution: distributed exclusively in South East Asia, except only one record known from Poland (Wang and Wang 2020, Gan et al. 2022, World Spider Catalog 2023). *Pancorius* currently includes 42 described species, with 11 species recorded from China, nine of which are endemics (Wang and Wang 2020, Gan et al. 2022, World Spider Catalog 2023).

The genus *Pancorius* remains inadequately studied because: more than half of the species (26) are known from a single sex (15 from males, 11 from females) (World Spider Catalog 2023); original descriptions are rather brief or illustrations are inadequate for many species, seven species cannot be identified due to the lack of diagnostic illustrations (Gan et al. 2022, World Spider Catalog 2023); the genus has never been revised at a global or large regional scale (Gan et al. 2022, World Spider Catalog 2023); and the diversity of this genus is still insufficiently known. However, in spite of the deficiencies just mentioned, most of the *Pancorius* species from China and neighbouring countries (such as Vietnam, India, Nepal etc.) have been well-studied, especially several new species described in recent years (Jastrzębski 2011, Logunov and Jäger 2015, Caleb et al. 2019, Wang and Wang 2020, Kanesharatnam and Benjamin 2021, Logunov 2021, Gan et al. 2022, Hoang et al. 2022). These species have been described in detail, alongside high-quality illustrations, to allow easy species recognition.

**Figure 1.**
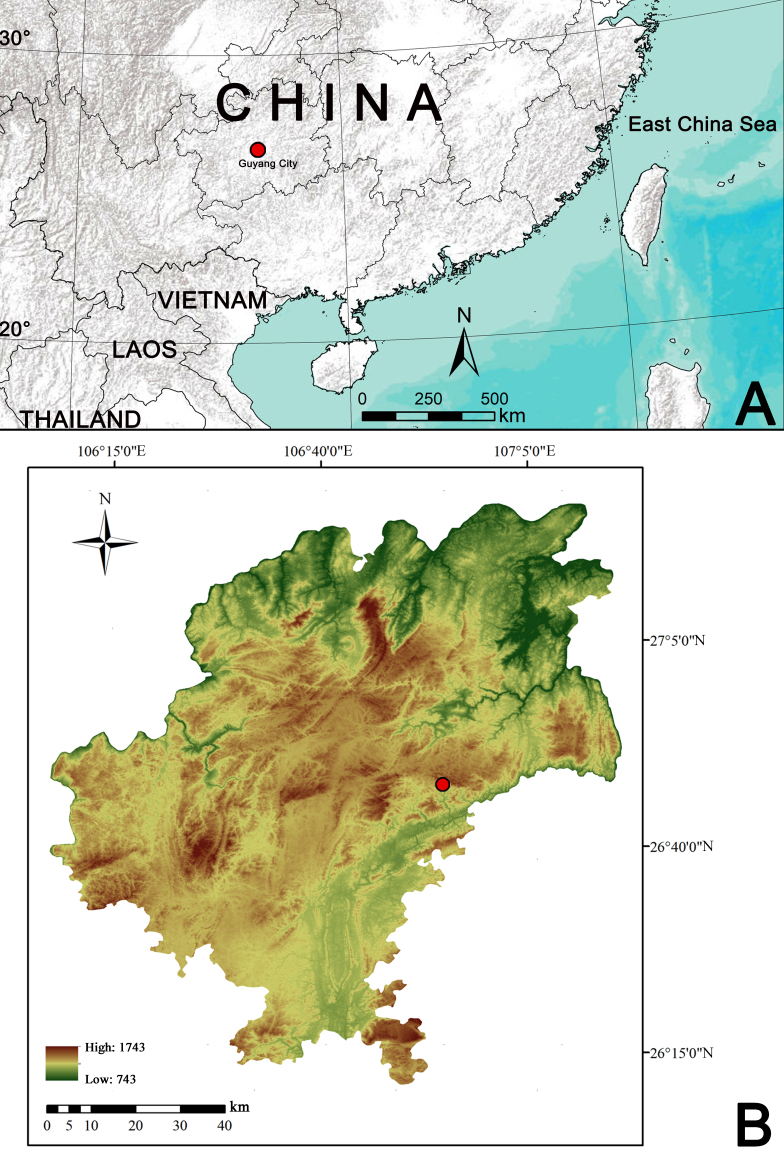
Distribution record of *Pancoriusguiyang* sp. nov. (red circles). **A** Locality of Guiyang City in China; **B** map of Guiyang City, showing type locality of the new species, Wudang District, Xiangzhigou scenic spot.

**Figure 2.**
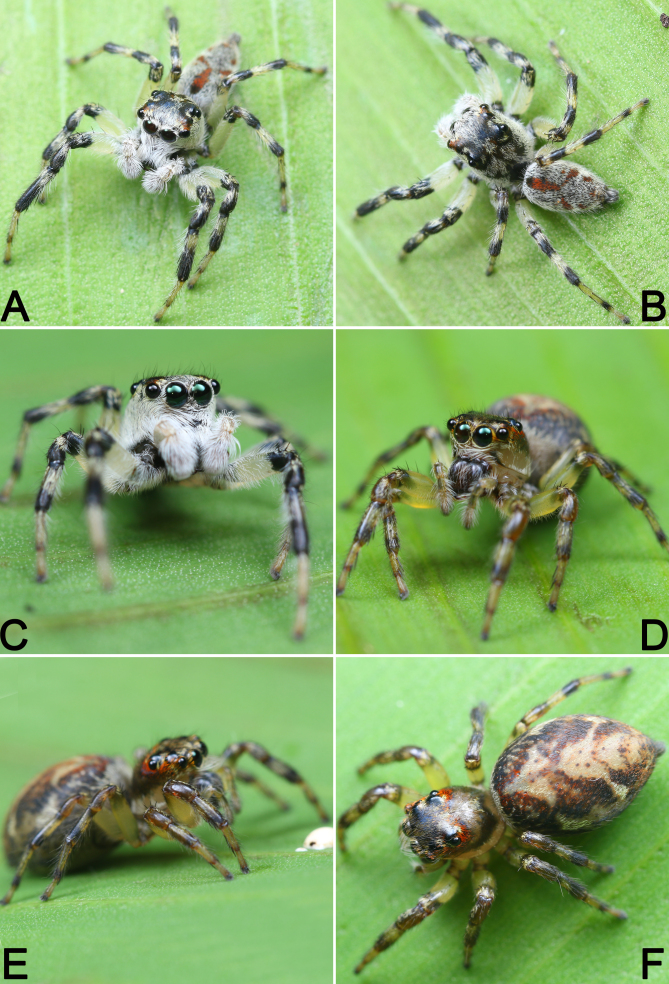
*Pancoriusguiyang* sp. nov., live specimens. **A**–**C** male holotype; **D**–**F** female paratype. Photographs by Qianle Lu (Shenzhen, Guangdong).

While examining spiders collected from Guiyang City, Guizhou Province, south-western China (Fig. 1), we found pairs of specimens of jumping spiders in the same location. Though dimorphism is exhibited by different sexes, mostly related to different colours and abdominal patterns (Figs 2, 5), we matched the female and male together, based on DNA barcoding data. Additionally, both sexes possess certain characters associated with the genus *Pancorius*, but can be easily distinguished from the other *Pancorius* species. This species is new to science and is described under the name of *Pancoriusguiyang* Yang, Gu & Yu, sp. nov. The aim of the current paper is to describe the new species, providing detailed morphological descriptions and illustrations.

## Materials and methods

Specimens in this study were collected by hand collecting from leaf-litter in Xiangzhigou scenic spot, Guiyang, Guizhou. Spiders were fixed and preserved in 95% ethanol. Specimens were examined with an Olympus SZX7 stereomicroscope; details were studied with an Olympus CX41 compound microscope. Female epigyne and male palp were examined and illustrated after being dissected. Epigyne was removed and cleared in warm lactic acid before illustration. Vulva was also imaged after being embedded in Arabic gum. Photos were made with a Cannon EOS70D digital camera mounted on an Olympus CX41 compound microscope. The digital images were taken and assembled using Helifocus 6.80 software package.

A DNA barcode was also obtained for the species matching. A partial fragment of the mitochondrial cytochrome oxidase subunit I (CO1) gene was amplified and sequenced for two specimens, using the primers LCOI1490 (5’-GGTCAACAAATCATAAAGATATTG-3’) and HCOI2198 (5’-TAAACTTCAGGGTGACCAAAAAAT-3’). For additional information on extraction, amplification and sequencing procedures, see Kanesharatnam and Benjamin (2021).

All measurements were obtained using an Olympus SZX7 stereomicroscope and given in millimetres. Eye diameters are taken at widest point. The total body length does not include chelicerae or spinnerets length. Leg lengths are given as total length (femur, patella, tibia + metatarsus, tarsus). Most of the terminologies used in text and figure legends follow Gan et al. (2022).

The type specimens are deposited in the Museum of Guizhou Normal University, Guiyang, China.

## Taxon treatments

### Pancorius guiyang

Yang, Gu & Yu sp. nov.

09481E47-03AF-5C57-BD8D-B6D6DCDACCAC

551BF45F-63C1-4814-8C01-5951E0B63774

#### Materials

- **Type status:** Holotype. **Occurrence:** recordedBy: Qianle Lu; individualID: YHGY199; individualCount: 1; sex: male; lifeStage: adult; behavior: foraging; preparations: whole animal (ETOH); associatedSequences: GenBank: OR372102; occurrenceID: 643415AE-C790-5664-8241-9BFF85089072; **Taxon:** order: Araneae; family: Salticidae; genus: Pancorius; specificEpithet: guiyang; scientificNameAuthorship: Yang, Gu & Yu; **Location:** continent: Asia; country: China; countryCode: CHN; stateProvince: Guizhou; county: Guiyang City; locality: Xiangzhigou scenic spot; decimalLatitude: 26.78; decimalLongitude: 106.92; **Identification:** identifiedBy: Cheng Wang; dateIdentified: 2022-08; **Event:** samplingProtocol: by hand; samplingEffort: 10 km by foot; year: 2022; month: 6; day: 1; **Record Level:** basisOfRecord: PreservedSpecimen
- **Type status:** Paratype. **Occurrence:** recordedBy: Qianle Lu; individualID: YHGY200; individualCount: 1; sex: female; lifeStage: adult; behavior: foraging; preparations: whole animal (ETOH); associatedSequences: GenBank: OR372101; occurrenceID: 9CD77831-8341-5B15-B9EC-FC39ED3F29D3; **Taxon:** order: Araneae; family: Salticidae; genus: Pancorius; specificEpithet: guiyang; scientificNameAuthorship: Yang, Gu & Yu; **Location:** continent: Asia; country: China; countryCode: CHN; stateProvince: Guizhou; county: Guiyang City; locality: Xiangzhigou scenic spot; decimalLatitude: 26.78; decimalLongitude: 106.92; **Identification:** identifiedBy: Cheng Wang; dateIdentified: 2022-08; **Event:** samplingProtocol: by hand; samplingEffort: 10 km by foot; year: 2022; month: 6; day: 1; **Record Level:** basisOfRecord: PreservedSpecimen

#### Description

**Figure 3.**
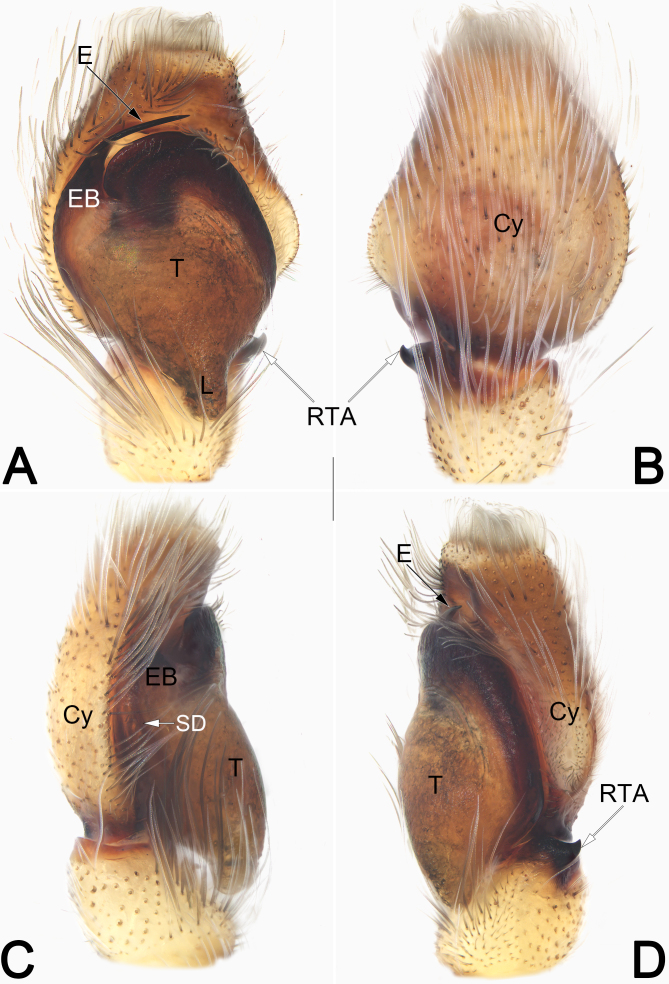
Male left palp of the holotype of *Pancoriusguiyang* sp. nov. **A** Ventral view; **B** Dorsal view; **C** Prolateral view; **D** Retrolateral view. Abbreviations: Cy = cymbium; E = embolus; EB = embolar base; L= lobe; RTA = retrolateral tibial apophysis; SD = sperm duct; T = tegulum. Scale bar: 0.2 mm (equal for **A–D**).

**Figure 4.**
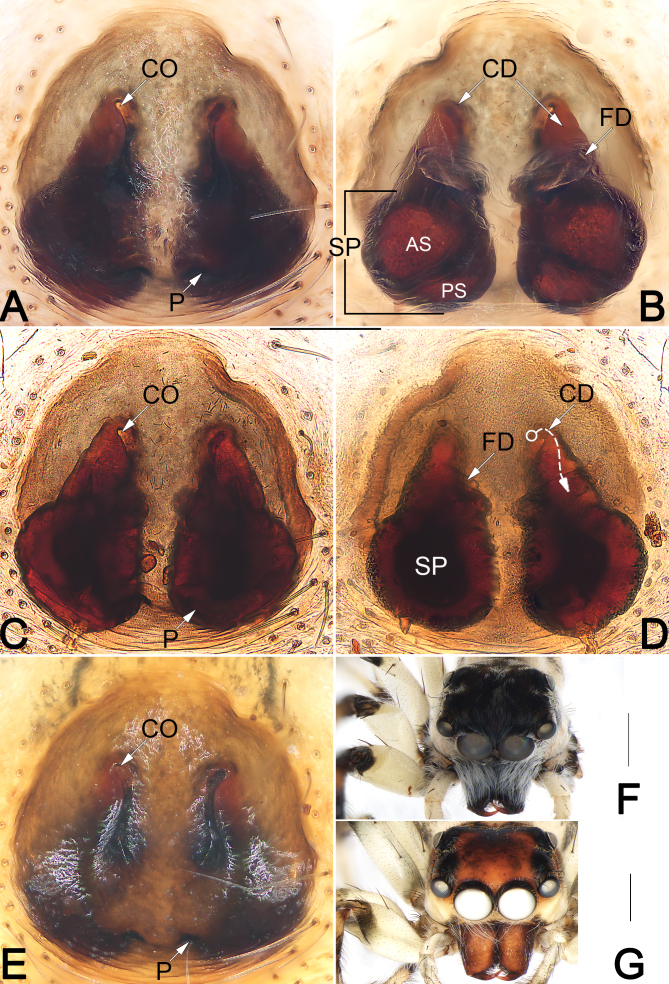
*Pancoriusguiyang* sp. nov., female paratype and male holotype, epigyne (**A–E**), frontal views of prosoma (**F**, **G**). **A**–**B** Macerated epigyne, ventral and dorsal; **C**–**D** Epigyne, macerated and embedded in Arabic gum, ventral and dorsal (dashed line in D showing schematic course of copulatory duct and connecting duct, dorsal); **E** Intact epigyne, ventral; **F** Male; **G** Female. Abbreviations: AS = anterior chamber of spermatheca; CD = copulatory duct; CO = copulatory opening; FD = fertilisation duct; P = epigynal pocket; PS = posterior chamber of spermatheca; SP = spermatheca. Scale bars: 0.2 mm (equal for **A–E**); 1 mm (**F, G**).

**Figure 5.**
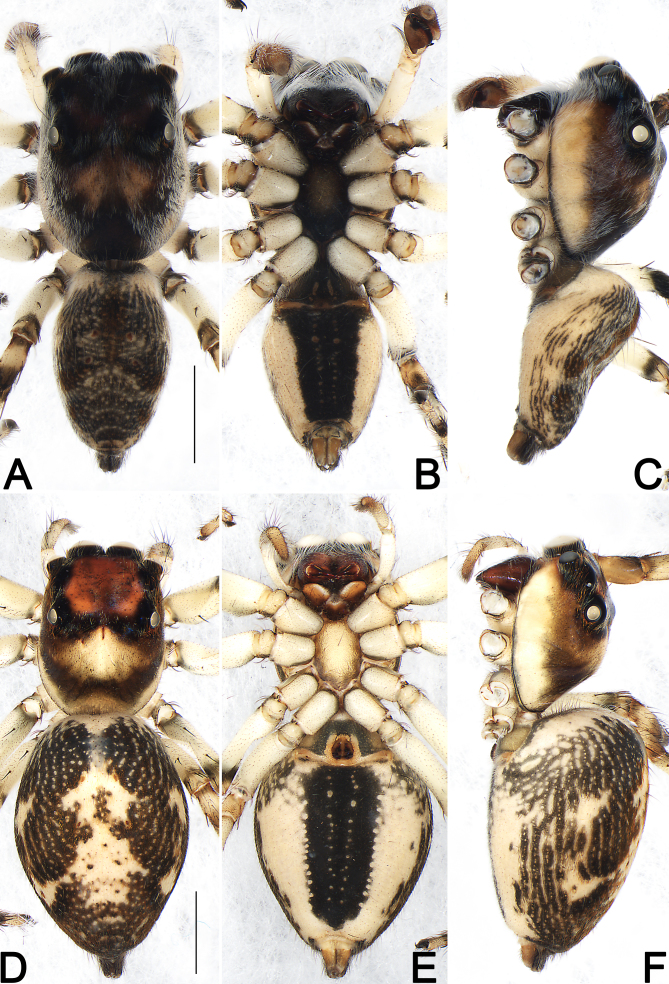
Habitus of *Pancoriusguiyang* sp. nov., male holotype (**A–C**) and female paratype (**D–F**). **A, D** Dorsal view; **B, E** Ventral view; **C, F** Lateral view. Scale bars: 2 mm (equal for **A–C**, equal for **D–F**).

**Description. Male** (holotype) (Fig. 2A–C, Fig. 3A–D, Fig. 4F, Fig. 5A–C). Dimensions in mm. Total length 7.87; carapace 4.02 long, 2.88 wide; abdomen 3.85 long, 2.35 wide. Eye sizes and interdistances: anterior median eyes (AME) 0.90, anterior lateral eyes (ALE) 0.48, posterior median eyes (PME) 0.10, posterior lateral eyes (PLE) 0.45; anterior eye row width (AERW) 2.77, posterior eye row width (PERW) 2.60, length of eye field (EFL) 2.02. Clypeal height 0.21. Sternum 1.71 long, 0.98 wide. Leg length: I 9.37 (2.55, 3.83, 1.95, 1.04), II 7.652 (2.37, 3.09, 1.30, 0.89), III 8.37 (2.67, 2.92, 1.86, 0.92), IV 9.10 (2.89, 3.19, 2.16, 0.86).

Living holotype male as in Fig. 2A–C, carapace was black, clothed with white hairs; abdomen covered with dense, white hairs, mottled with red patches; legs light yellow, all legs with conspicuous dark annuli in the distal parts of femur, patella and tibia.

Habitus in ethanol (Fig. 4F, Fig. 5A–C). Carapace black, broadened, marginally with dense, white hairs; cephalic region bearing a longitudinal, broad, distinct band of hairs, centrally with a pair of nearly triangular areas. Fovea indistinct, represented by a dark, longitudinal slit. Chelicerae deep dark, with two promarginal and one retromarginal teeth. Labium and endites basically coloured as chelicerae, endites depressed posteriorly, slightly convergent anteriorly, antero-inner margins white; labium nearly linguiform, anterior margin with sparse setae. Sternum light brown centrally, dark marginally, more or less shield-shaped. Abdomen elongate-oval in dorsal view, tapering posteriorly. Dorsum basically black, centrally with a narrow scutum extending ca. 1/2 of abdomen length, gradually narrowing posteriorly, with two pairs of inconspicuous muscular depressions on either side, followed by four ˄-shaped streaks, covered with dark thin hairs; venter pale yellow laterally, with a broad dark brown patch bearing a pair of dotted lines centrally.

Palp (Fig. 3A–D). Tibia short, nearly as wide as long, ca. 2/5 length of cymbium (Cy); retrolateral tibial apophysis (RTA) short, about 1/3 of tibia length, thumb-like, tip sharp and point antero-dorsally. Tegulum (T) elongate, oval and bulging, about 1.3× longer than wide; sperm duct (SD) indistinct in prolateral and ventral view, distinct in retrolateral view, forming a loop along tegular margin; tegulum with tapered posterior lobe extending downwards in ventral view, lobe (L) finger-like and located at approximately the 5–6 o’clock position. Embolar base (EB) represented by an enlarged tubercle, situated antero-prolateral of the tegulum (approximately 9–10 o’clock position); the free part of embolus (E) needle-shaped, strongly sclerotised, slightly curved medially and tapering at distal half to a pointed tip, terminating at ca. 12 o’clock position, apex directed towards about 1 o’clock position.

**Female** (Fig. 2D–F, Fig. 4A-E, G, Fig. 5D–F). Dimensions in mm. Total length 9.72; carapace 4.27 long, 3.08 wide; abdomen 5.94 long, 4.18 wide. Eye sizes and interdistances: AME 0.81, ALE 0. 51, PME 0.10, PLE 0.47; AERW 2.78, PERW 2.71, EFL 2.01. Clypeal height 0.20. Sternum 1.70 long, 1.05 wide. Leg length: I 7.69 (2.33, 3.17, 1.29, 0.90), II 6.94 (2.21, 2.83, 0.84, 1.06), III 8.66 (2.68, 3.07, 1.72, 1.19), IV 8.75 (2.69, 3.18, 1.84, 1.04).

One living paratype female as in Fig. 2D–F, carapace dark in front, yellowish-brown posteriorly and marginally, densely covered with orange setae around eye field; abdomen basically black, centrally with large, yellowish-white patterns, sparsely mottled with red hairs; legs light yellow, all legs with conspicuous reddish-brown annuli in the distal parts of femur, patella, tibia and metatarsus.

Colour in ethanol Fig. 4G, Fig. 5D–F). Carapace red-brown to yellowish-brown; eye field red-brown centrally, black marginally; thorax yellowish-brown, with a black, nearly W-shaped transverse band. Dorsum of abdomen with a longitudinal yellowish-white band consisting of two arrow-shaped patterns, ca. 2/3 length of abdomen. Other characters as in holotype male, but distinctly larger in size.

Epigyne (Fig. 4A–E). Epigynal plate slightly longer than wide, margin distinctly delimited; spermathecae (SP) clearly visible through the tegument in ventral view. Copulatory openings (CO) longitudinal, slit-shaped, anteriorly widened. Paired epigynal pockets represented by two clefts in which is situated the posterior margin of epigynal plate, small and nearly triangular, separated from each other by ca. 1× their width. Copulatory ducts (CD) short, proximally slender, extending and widening posteriorly, descending obliquely, finally connecting with posteriorly located spermathecae. Spermathecae (SP) large, almost round, obviously separated from each other about 1/2 their diameter; spermatheca divided into two oval chambers. Fertilisation duct (FD) membranous and lamellar, large, ca. 2/3 of spermathecal diameter, originating from the antero-inner surface of anterior chamber of spermatheca, anterolaterally extending.

##### DNA barcodes

5'GGTGCTTGAGCTGCTATAGTAGGAACTGCAATAAGAGTATTAATTCGTATAGAATTGGGGCAAACTGGGAGATTTTTAGGCAATGAACATTTATATAATGTAATTGTTACAGCACACGCATTTGTAATAATTTTTTTTATAGTAATACCTATTTTAATTGGAGGATTTGGTAATTGATTAGTCCCTTTAATGTTGGGAGCGCCTGATATGGCTTTTCCTCGAATAAATAATTTGAGATTTTGATTATTACCTCCTTCTTTGATTTTGTTATTTATTTCTTCTATGGCTGAAATGGGAGTGGGAACAGGATGAACTGTTTATCCACCTTTAGCATCTATTGTAGGACATAATGGTAGTTCTGTGGATTTTGCTATTTTTTCTTTACATTTAGCTGGTGCTTCTTCTATTATAGGAGCTATTAATTTTATTTCAACTGTAATTAATATACGATCGGTAGGTATAACTTTGGATAAAGTTTCTTTATTTGTATGATCAGTTATTATTACTACTGTATTATTATTATTATCATTACCTGTGTTGGCGGGTGCTATTACTATATTATTGACAGATCGTAATTTTAATACTTCTTTTTTTGATCCAGCAGGTGGAGGAGATCCTATTTTATTTCAACATTTATTTTGATTTTTTG3' (holotype, YHGY199; Genebank: OR372102)

5'GGTGCTTGAGCTGCTATAGTAGGACTGCAATAAGAGTATTAATTCGTATAGAATTGGGGCAAACTGGGAGATTTTTAGGCAATGAACATTTATATAATGTAATTGTTACAGCACACGCATTTGTAATAATTTTTTTTATAGTAATACCTATTTTAATTGGAGGATTTGGTAATTGATTAGTCCCTTTAATGTTGGGAGCGCCTGATATGGCTTTTCCTCGAATAAATAATTTGAGATTTTGATTATTACCTCCTTCTTTGATTTTGTTATTTATTTCTTCTATGGCTGAAATGGGAGTGGGAGCAGGATGAACTGTTTATCCACCTTTAGCATCTATTGTAGGACATAATGGTAGTTCTGTGGATTTTGCTATTTTTTCTTTACATTTAGCTGGTGCTTCTTCTATTATAGGAGCTATTAATTTTATTTCAACTGTAATTAATATACGATCGGTAGGTATAACTTTGGATAAAGTTTCTTTATTTGTATGATCAGTTATTATTACTACTGTATTATTATTATTATCATTACCTGTGTTGGCGGGTGCTATTACTATATTATTGACAGATCGTAATTTTAATACTTCTTTTTTTGATCCAGCAGGTGGAGGAGATCCTATTTTATTTCAACATTTATTTTGATTTTTTG3' (paratype, YHGY200; Genebank: OR372101)

#### Diagnosis

The male of this new species closely resembles that of *P.crinitus* Logunov & Jäger, 2015 from Vietnam and *P.candidus* Wang & Wang, 2020 from China. The three species share the similarly distinctly short RTA whose apex points dorsally (vs. RTA relatively longer and apex pointing anteriorly in all other congeners). However, *P.guiyang* sp. nov. can be differentiated from *P.crinitus* and *P.candidus* by the distinctly slender, needle-shaped embolus without subdistal projection (Fig. 3A) (vs. embolus thicker and claw-shaped in *P.crinitus* as in Logunov and Jäger (2015): fig. 40, with a subdistal projection in *P.candidus* as in *Wang and Wang (2020)*: figs. 5–7). The female of *P.guiyang* sp. nov. also resembles that of *P.crinitus* in having similar shape of the vulva, but can be separated by the paired epigynal pockets distinctly concaved, narrowed (vs. very shallow and wide) (cf. Fig. 4A, C, E and Logunov and Jäger (2015): fig. 44) and by copulatory ducts descending slightly oblique (vs. running distinctly oblique, almost horizontal) (cf. Fig. 4B, D and Logunov and Jäger (2015): fig. 43).

#### Etymology

The species name is derived from the name of the type locality; noun in apposition.

#### Distribution

Known from the Guiyang City, Guizhou Province, China (Fig. 1).

#### Biology

*Pancoriusguiyang* sp. nov. is a typical leaf-dwelling spider, the types inhabit bamboo forest close to a small stream in the core zone of Xiangzhigou scenic spot and were collected by beating twigs and branches.

## Supplementary Material

XML Treatment for Pancorius guiyang
